# Supplementary material for: The doodle dilemma: How the physical health of ‘Designer-crossbreed’ Cockapoo, Labradoodle and Cavapoo dogs’ compares to their purebred progenitor breeds
Source: PLoS One. 2024 Aug 28;19(8):e0306350. doi: 10.1371/journal.pone.0306350 (PMC11355567; doi:10.1371/journal.pone.0306350)
Supplement: S1 Text — (DOCX) [file pone.0306350.s001.docx]

**Exploring ownership experiences of popular dog breeds and their crosses in the UK**

**What is this research about?**

Around 1 in 3 households in the UK own a dog, with dog ownership recently booming during the Pandemic. Within the UK dog population, there is a wide range of over 200 breeds and their crosses. Understanding of if and how experiences of owning specific breeds or crossbreeds differs is poorly understood. This study aims to explore various aspects of dog ownership, including daily caregiving, experiences of dog health and behaviour, relationships between dogs and owners, and how these aspects met, or differed from owner expectations of their dog while they were a puppy. This study focuses on the ownership of several popular dog breeds and crossbreeds in the UK. By taking part, your views will help us better understand your experience as a dog owner of your particular breed/crossbreed and consequently, enable vets, behaviourists and animal welfare organisations to tailor advice and support for owners in the future.

**Who is eligible to take part in this study?**

Owners who ***currently*** own at least one of the following breeds/crossbreeds are eligible for this study:

*N.B ALL dogs within each breed are welcome to participate including those from working, show and pet lines*

- Cavalier King Charles Spaniel
- Cavapoo (Cavalier King Charles Spaniel crossed with any Poodle breed)
- Cockapoo (Cocker Spaniel crossed with any Poodle breed)
- Cocker Spaniel
- Labradoodle (Labrador Retriever crossed with any Poodle breed)
- Labrador Retriever
- Miniature Poodle
- Standard Poodle
- Toy Poodle

If you own more than one dog, please fill out the following survey with the dog whose name comes FIRST in the alphabet. This survey is to be complete for ONE dog per household only.

Owners must meet **all** of the following criteria:

- Are over 18 years of age.
- Resident of the United Kingdom
- Own one of the above breeds/crossbreeds

**How do I take part?**

To take part, you simply need to answer a series of questions online as part of this survey. The survey will be open until 28^th^ February 2023

Completion of the entire survey takes around 25 minutes of your time but can contribute hugely to a lifetime of improved welfare for dogs in the future.

**What will happen to my information?**

Your information will be kept by the RVC in accordance with GDPR. Only the scientists undertaking this study (Ms Gina Bryson, Dr. Rowena Packer, Dr. Dan O’Neill, Dr. Claire Brand, Dr Zoe Belshaw and Dr Fiona Dale) will have access to your data. These data will be anonymised and used for research and only anonymised results and data will be published in a peer-reviewed veterinary journal. Publications may include quotes from your written responses (if applicable), but these will be anonymised, and you will not be identifiable from any quotes.

**Can I change my mind and withdraw my data?**

Should you wish to withdraw your data from the study at any point, please contact either Gina Bryson (gbryson19@rvc.ac.uk) or Dr. Rowena Packer and Dr. Claire Brand (pandemicpuppies@rvc.ac.uk). Any data that you request to be withdrawn will be permanently deleted.

**What if taking part raises concerns?**

If this study raises any concerns about your dog's welfare, then please contact a veterinary surgeon to discuss. Links on how to find a veterinary surgeon and/or animal behaviour professional in your area are included at the end of the survey. Resources on specific canine welfare issues raised will also be included at the end of the survey.

If this study raises any concerns about your own mental health, then please access these NHS resources: [www.nhs.uk/conditions/stress-anxiety-depression/mental-health-helplines/](http://www.nhs.uk/conditions/stress-anxiety-depression/mental-health-helplines/)

**Consent Questions and Inclusion Criteria**

**Q1. I confirm that**

I am over 18 years of age

I am a resident of the UK

I have read and understood the above information and give consent for my answers to be used for this research study and any resulting publications

**PAGE ONE: ESSENTIAL QUESTIONS**

**Q1. Please select the breed or crossbreed of dog you are answering about.**

*N.B ALL dogs within each breed are welcome to participate including those from working, show and pet lines*

Drop down list:

Cavalier King Charles Spaniel [logic - goes to question b]

Cavapoo (Cavalier King Charles Spaniel crossed with any Poodle breed) [logic - goes to question 1a]

Cockapoo (Cocker spaniel crossed with any Poodle breed) [logic - goes to question 1a]

Cocker Spaniel [logic - goes to question 1c]

Labradoodle (Labrador Retriever crossed with any Poodle breed) [logic - goes to question 1a]

Labrador Retriever [logic - goes to question 1c]

Miniature Poodle [logic - goes to question 1c]

Standard Poodle [logic - goes to question 1c]

Toy Poodle [logic – goes to question 1c]

Not on the list (please specify) **[free text]**

**1 a) If known, what type of Poodle is in your dog’s ancestry (parents and grandparents)?**

**(N.B please select ALL that apply, if more than one Poodle breed is involved in your dog’s ancestry)**

Toy Poodle

Miniature Poodle

Standard Poodle

I don’t know/I’m not sure

**1 b) If known, what type or ‘generation’ of cross is your dog (i.e. where in their ancestry was their non-Poodle parent breed mixed with a Poodle)?**

- I don’t know/I’m not sure
- F1 cross (e.g. their parents were a purebred Poodle (Toy/Miniature/Standard) and a purebred second breed (e.g. a Cavalier King Charles Spaniel, Cocker Spaniel, Labrador Retriever))
- F1B (eg. Their parents were an F1 cross and a purebred Poodle (Toy/Miniature/Labrador))
- F1BB (eg. Their parents were an F1B cross and a purebred Poodle (Toy/Miniature/Standard))
- F2 (eg. Their parents were both F1 crosses)
- F2B (eg. Their parents were an F2 cross and a purebred Poodle (Toy / Miniature / Standard))
- F2BB (eg. Their parents were an F2B cross and a purebred Poodle (Toy / Miniature / Standard))
- F3 (eg. Their parents were both F2 crosses)
- Multigeneration (eg. Their parents were both a cross of any generation beyond F3)

**1c) Is your puppy/dog registered with The UK Kennel Club?**

Yes

I’m not sure

No

No – registered with another canine registration body, e.g. a working dog registry (please specify) **[free text]**

**Q2. What is your puppy’s/dog’s date of birth? Please enter in the format DD/MM/YY**

*N.B. If you are unsure, please leave relevant part as ‘00’*

Date text field

01/01/01

**Q. Is your puppy/dog female or male?**

Male

Female

**Q. Is your dog neutered?**

Yes

No

I don’t know

**Q Is your puppy/dog insured?**

Yes

No – and I do not plan to insure them

No – but I plan to insure them in the future

No – they were insured but I have since cancelled or did not renew their policy

No – I have never heard of pet insurance

No – other **[free text]**

**PAGE 2: Core Demographic Questions (For all Owners)**

**Q. Are you the primary carer for your puppy/dog (i.e. the person in your household that provides your puppy/dog with the majority of care such as feeding and walking)?**

Yes

No

I share the role of primary carer for my puppy/dog with someone else in the household

I share the role of primary carer for my puppy/dog with someone else in a different household

N/A – I no longer have my puppy/dog

**Q. How old are you?**

18 to 24 years old

25 to 34 years old

35 to 44 years old

45 to 54 years old

55 to 64 years old

65 to 74 years old

75 years old or older

**Q. What is your gender?**

Female

Male

Other

Prefer not to say

**Q. If you are happy to, please provide the first three/four digits of your postcode (e.g. AL9)**

[free text]

**Q. Are you or any member of your household employed in the canine and/or animal care sector (e.g. veterinary nurse, dog groomer, dog trainer, etc.)?**

Yes

No

I’m not sure

**Q. Which canine and/or animal care sector are you or a member of your household employed in?**

Please select **all** options that apply

Veterinary surgeon

Veterinary nurse

Animal care assistant

Veterinary scientist

Dog trainer

Dog behaviourist

Dog groomer

Rehoming centre staff

Canine physiotherapist

Animal insurance provider

Dog day care/boarding kennels

Dog walker

Academic sector (e.g. university staff and students that are part of an animal care/behaviour course)

Allied veterinary professionals (e.g. physiotherapist, hydrotherapist, receptionist at a veterinary practice)

Animal care sector (e.g. pet shop staff, animal welfare officer)

Non-canine animal professional (e.g. equine, farm and zoo industry)

Other (please specify)

**Q. Did you grow up with a dog in your childhood home?**

Yes

No

**Q. As an adult, have you ever owned or co-owned a dog before you purchased your puppy/dog?**

Yes

No – but someone else in my household has

No – I am/everyone in my household is a first-time dog owner(s)

**Owners who have not completed the PP survey:**

**Did you or your household carry out any research into owning dog and/or which breed/crossbreed to buy before you purchased your puppy?**

1. No
2. No – but I am already an experienced dog owner
3. Yes – I carried out research before purchasing my puppy **[survey logic – go to b]**

**b) What sources of information did you or your household use when researching dog ownership and/or which breed/crossbreed to buy prior to buying your puppy?**
Please select **all** options that apply
The Kennel Club website
An animal charity website, e.g. Dogs Trust, RSPCA, PDSA, etc.
A breed/crossbreed-specific online resource (e.g. website/forum)
Social media sites, e.g. Facebook, Instagram
Book(s)
Dog-specific magazine(s)
My veterinary professional (e.g. veterinary surgeon, veterinary nurse)
Talking to friends or family who own or had owned a dog
Talking to a dog breeder

Talking to a non-veterinary animal professional (e.g. trainer, behaviourist)

I am already an experienced dog owner but purchased a new breed

I have professional experience with dogs (veterinary and non-veterinary)

Talking to current dog owners that I met (not friends or family)

Seeking practical experience of caring for dogs (e.g. Borrow My Doggy/dog sitting, fostering, etc.)

Other digital media sources (internet searches/sites, TV, DVD, etc.)
I can’t remember

None of these options

Other [free text]

**c) What characteristics were you looking for in a dog when selecting a particular breed/crossbreed to buy?** Please select **all options** that apply

I’ve owned this breed or crossbreed before

I grew up with or had childhood experiences with this breed/crossbreed

Friends or family currently own this breed/crossbreed

Affordable purchase cost of puppies

Affordable cost of upkeep

Appearance/looks

Low grooming needs

Low exercise requirements

Good with children

Good companion

Size suited to my lifestyle

Generally healthy breed/crossbreed

Popularity of the breed/crossbreed

Working ability of the breed/crossbreed

Long life expectancy

Exercise encouragement

Celebrity/Influencer endorsement/ownership

Hypoallergenic

Easy to train

None of these options – I did not have any specific characteristics I was looking for

None of these options – someone else in the household selected the breed/crossbreed of our puppy

Other perceived temperament/personality traits of the breed/crossbreed

Low or non-shedding breed/crossbreed

Specific genetic characteristics of the breed/crossbreed

I’ve always wanted to own this breed/crossbreed

Other [free text]

**What characteristics were you looking for in a breeder?**

Please select **all options** that apply

Availability of the breed I wanted

Availability of puppies at the time I wanted

Bred the colour of the breed/crossbreed I wanted to purchase

Reasonably priced puppies

Lived within the distance I was willing to travel

Good communication with me

They performed health tests for the breed/crossbreed I wanted

Someone I felt was trustworthy

Someone I felt cared for their dogs

They would allow me to see the puppies’ mother

They would allow me to see the puppies’ father

They registered their puppies with The Kennel Club

The dogs they bred from had been awarded prizes at dog shows

A member of the Kennel Club Assured Breeder Scheme

A council registered breeder

Someone I already knew

They bred dogs with specific working and/or sporting characteristics

They were a member of a specific breed/crossbreed club/association

They registered their puppies an international canine registration body (e.g. FCI)

Other (please specify) **[free text]**

**How did you find the breeder of your puppy/dog?**

Please select **all options** that apply

A general selling website, e.g. FreeAds, Gumtree, Preloved

An animal specific selling website, e.g. Pets4Homes, Champdogs

The Kennel Club website ‘Find A Puppy’ search

The breeder’s website

The breeder’s social media account

A social media breed/crossbreed-specific group

Local newspaper advert

Dog specific magazine(s)/newspaper(s)

An advert in a local shop

I already knew the breeder (e.g. colleague, friends, family, repeat purchase)

Recommendation from a friend

Recommendation from another breeder/stud dog owner

Recommendation from someone who is not a colleague, friend, family or animal professional

Recommendation from someone who is an animal professional (e.g. veterinary surgeon, veterinary nurse, dog trainer, etc.)

Recommendation from a stranger after a chance encounter

An advert seen in another online location (including general internet searches and social media)

A physical advert in another location (e.g. vets noticeboard)

The breeder contacted me directly following expression of interest for a puppy

Other **[free text]**

**On the day you brought your puppy home, which, if any other dogs did you see your puppy with?**

Please select **all options** that apply

Their littermates

Other puppies (unsure if they were littermates)

Their mother

Their father

Another dog(s) they were not related to (e.g. another breed)

I only saw my/our puppy

I don’t remember

I’m not sure, I wasn't the person who collected my/our puppy

Another adult dog(s) they were related to (e.g. aunts, grandparents, older siblings)

Another adult dog(s) the breeder claimed were my puppy’s parent(s), but I’m not sure

Other puppies they were related to (but not littermates)

Other puppies they were not related to (e.g. another breed)

Other (please specify) **[free text]**

**How old were you told your puppy was when you brought them home?**

Under 6 weeks old

7 weeks old

8 weeks old

9weeks old

10 weeks old

11 weeks old

12 weeks old

13 weeks old

14 weeks old

15 weeks old

16 weeks old

I’m not sure/can’t remember

**How much did you pay in total to purchase your puppy/dog?**

*N.B. Please only include the price of your puppy and not any associated purchases, e.g. food, collar, bowls, etc.*

£ (please state a whole number in pounds, do not include the pound sign) **[Number box]**

Prefer not to say

I can’t remember

**Had your breeder provided your puppy with any of the following prior to you taking them home?**

Please select **all options** that apply

Worming treatment

Flea treatment

Health check by a vet

Microchip

First vaccinations

Second vaccinations

The Puppy Contract

The Puppy’s Passport

**Did you ask your breeder to see any information related to health testing of your puppy’s parents?** *N.B. health tests are not available for all dog breeds*

*Columns*

Yes, and they provided me with it

Yes, but they couldn’t provide it

No, I did not ask about this

No, I did not ask but it was provided

No, I do not believe there are any tests available for my puppy’s breed/crossbreed

*Rows*

The results of DNA (genetic) tests

The results of veterinary screening tests (e.g. hips, elbows, knees, eyes, respiratory testing)

***PAGE 3: Health***

*In this section, you will be asked questions related to your dog’s health and well-being, medical conditions and diagnoses and any treatment your dog has received.*

**Q. Is your puppy/dog registered with a vet?**

Yes

No

Not yet, but I intend to in the future

**Q. Has your dog shown any of the following in the past year, regardless of whether you sought veterinary advice or not?** Please select **all options** that apply.

*Columns*

1. No
2. Yes, but I did not seek veterinary advice/attend a veterinary appointment
3. Yes, and I sought veterinary advice/ attended a veterinary appointment
4. I’m not sure/I can’t remember

*Rows*

1. Runny and/or red eye(s)
2. Diarrhoea and/or runny faeces
3. Vomiting (being sick)
4. Worms in faeces
5. Fleas/other parasites visible in fur/on skin
6. Hair loss
7. Wounds/sore areas of skin
8. Frequent itching/licking
9. Coughing
10. Sore or infected ear(s)
11. Problems with their anal glands
12. Dental problems
13. Lameness
14. Overgrown nails
15. Being overweight
16. Losing weight
17. Arthritis
18. Lumps
19. Funny turns (suspected/confirmed to be fits/seizures)
20. Urinary incontinence (leaking urine)
21. Breathing problems
22. Eating non-food items (e.g., cloth, plastic, stones, faeces)

**Q. Other than the health problems listed in the previous question, has your dog experienced any other health concerns in the past year? Please describe in your own words, including whether you sought veterinary advice/attended a veterinary appointment for each issue described**

**……………………………………………………………………………………………………………………………………………………………………………………………………………………………………………………………………………………**

**Q. Has your dog ever been professionally diagnosed with any of the following**

**(**Please select **all options** that apply**):**

Addisons disease (adrenal gland hormonal disorder)

Paroxysmal dyskinesia (episodic movement disorder)

Epilepsy (seizures)

Hip and/or elbow dysplasia (joint abnormality)

Patella Luxation (kneecap moves out of position)

Von Willebrand’s Disease (bleeding disorder)

Cruciate ligament rupture (tearing of knee tissue)

Cancers

None of the above

**How much, roughly, have you spent, in the past year, on non-routine treatment and appointments for your dog at a veterinary clinic? (non-routine does not include vaccinations, health checks or neutering)**

I have never taken my dog to the vets

<£200

£200 - £500

£500 - £1000

£1000 - £2000

£2000 - £5000

£5000 - £10000

>£10000

I don’t know

**How healthy would you rate your dog in general? [RATING SCALE]**

Worst health possible

Very poor health

Poor health

Moderately poor health

Moderately good health

Good health

Very good health

Best health possible

**How would you rate the health of your dog compared to the rest of their breed/ crossbreed?**

Much less healthy than average for the breed/crossbreed

Less healthy than average for the breed/crossbreed

Average health for the breed/crossbreed

Healthier than average for the breed/crossbreed

Much healthier than average for the breed/crossbreed

**Have your expectations of health been met for your breed/crossbreed?**

1. Worse than I expected
2. As I expected
3. Better than I expected
4. I’m not sure/I can’t remember

**PAGE 4: Dog Routine and Care**

*The following sections will be asking you about the upkeep and maintenance of your dog and how this affects you and your household.*

***Section 1: Grooming and general upkeep***

**QHow often do you groom/brush your own dog yourself?**

Every day

Every 2-3 days

Every week

Fortnightly

1-2 times a month

Less than once a month

I don’t groom/brush my dog

I don’t know

**Q.Is the number of times you groom your own dog more/less/same as expected?**

More than expected

The same as expected

Less than expected

I don’t know

**Q. How often do you currently use professional dog grooming services for your dog?**

I have never used professional grooming services

I used to use professional dog grooming services but don’t anymore [logic – go to b)]

More than once a month

Every month

Every two months

Every 3-5 months

Every 6 months

Less than every 6 months

I don’t know

**b) What was the reason for stopping the grooming service? Please select all that apply**

It was too expensive

My dog reacted badly to being groomed (eg. fearful or aggressive to groomer) so we chose not to continue

Grooming services were challenging to access (E.g. services are s too far away, limited appointments available)

We were refused service due to our dog’s behaviour

We were disappointed with the service e.g. our dog’s appearance was not what we expected

Would rather not say

Other (go to c))

**c)Please explain in your own words your reason for stopping grooming services [Free text]**

**Q) On average, how much do you spend on professional grooming for your dog every six months?**

< £50 every six months

£50 - £74 every six months

£75 - £99 every six months

£100 - £150 every six months

>£150 every six months

**Q) Was the cost of the grooming more or less than you expected before purchase of your dog?**

N/A – my dog has never been professionally groomed

Considerably more than I expected

Slightly more than I expected

The same as I expected

Slightly less than expected

Considerably less than expected

**Has/will the rising cost-of-living affect how often or whether your dog is professionally groomed?**

N/A – my dog has never been professionally groomed

Yes, my dog is/will be professionally groomed less often

Yes, my dog is/will be professionally groomed more often

No change

**QHow often do you clean your dog’s ears?**

Once a week

Every Fortnight

Every Month

Every 2-3 months

Every 4-6 months

I don’t know/can’t remember

I don’t clean my dog’s ears

**Q.Is the number of times you clean your dog’s ears more/less/same as expected?**

More than expected

The same as expected

Less than expected

I don’t know

**QHow often do you bathe your dog?**

Every month

Every 2-3 months

Every 4-6 months

Once a year

I don’t know/can’t remember

I don’t bathe my dog

**Q.Is the number of times you bathe your dog more/less/same as expected?**

More than expected

The same as expected

Less than expected

I don’t know

**QHow often do you trim your dog’s nails?**

More than 1-2 times a month

1-2 times a month

Every 2-3 months

Every 4-6 months

Once a year

I don’t know/can’t remember

I don’t trim my dog’s nails

**Q.Is the number of times you trim your dog’s nails more/less/same as expected?**

More than expected

The same as expected

Less than expected

I don’t know

**Section 2: Your household**

**Do you, or any member of your household, suffer from allergies?**

***(allergies refer to a reaction to substances such as pollen, animal dander and certain foods that result in a wide range of symptoms including, but not limited to, rashes, a runny nose, itchiness and breathlessness)***

Yes – 1 household member [go to Qa]

Yes – more than 1 household member (go to Qb)

No (go to Q)

**a) If yes, since purchasing your dog, have the allergy symptoms of your affected household member….**

Increased

Decreased

Stayed the same

I’m not sure

I’d rather not say

**b) If yes, since purchasing your dog have the allergy symptoms of your affected household members….**

Increased for all affected people

Increased for at least one affected person

Decreased for all affected people

Decreased for at least one affected person

Stayed the same for all affected people

Stayed the same for at least one affected person

I’m not sure

I’d rather not say

**Do you, or any member of your household, suffer from asthma?**

***(asthma refers to a lung condition that can cause breathing difficulties including breathlessness, wheezing and coughing*)**

Yes – 1 household member (go to Qa)

Yes – more than 1 household member (go to Qb)

No (go to Q)

**a) If yes, since purchasing your dog, have the asthma symptoms of your affected household member….**

Increased

Decreased

Stayed the same

I’m not sure

I’d rather not say

**b) If yes, since purchasing your dog have the asthma symptoms of your affected household members….**

Increased for all affected people

Increased for at least one affected person

Decreased for all affected people

Decreased for at least one affected person

Stayed the same for all affected people

Stayed the same for at least one affected person

I’m not sure

I’d rather not say

**Do you believe any dog breeds/crossbreeds can be hypoallergenic (i.e. less likely to cause an allergic reaction in humans?**

Yes [logic – go to next question]

No

I’m not sure

**When choosing a breed of dog, did you consider whether their breed/crossbreed was considered to be hypoallergenic or not an important element of your selection process?**

Yes, choosing a hypoallergenic breed was the most important factor in our breed/crossbreed selection [logic – go to a]

Yes, choosing a hypoallergenic breed was one of a number of important factors we considered in our breed/crossbreed selection [logic – go to a]

No, whether a breed/crossbreed was considered to be hypoallergenic or not was not important in our breed/crossbreed selection

Other (please describe in your own words)

1. **Have your expectations of hypoallergenicity been met for your breed/crossbreed?**

Yes, I and/or a member of the household have not experienced exacerbation/increase in any allergy-like symptoms since owning the dog, as expected

No, I and/or a member of the household have experienced exacerbation/increase in allergy-like symptoms since owning our dog, which did not meet my/our expectations for hypoallergenicity [go to b]

I’m not sure

I’d rather not say

**b) Because of the unexpected exacerbation/increase in allergy-like symptoms in your household, are you or considering or have you ever considered rehoming your dog?**

Yes, I am likely to rehome my dog in the future due to this

Yes, I have considered rehoming my dog due to this, but decided against it

No, I have not considered rehoming my dog due to this

Other

**Section 3: Owner experience**

**Please read the following statements and consider how frequently you feel this way regarding your dog, if ever:**

**How often do you feel that looking after your dog is a chore?**

Once a day

Once a week

Once a month

Once a year

Never

**How often do you feel that having a dog is more trouble than it is worth?**

Once a day

Once a week

Once a month

Once a year

Never

**How hard is to look after your dog?**

Very hard

Hard

Neither hard nor easy

Easy

Very Easy

**How often does your dog stop you doing things you want to?**

Once a day

Once a week

Once a month

Once a year

Never

**Please read the following statements and consider how much you agree with each, if at all:**

**It is annoying that I sometimes have to change my plans because of my dog**

Strongly agree

Agree

Neither Agree nor Disagree

Disagree

Strongly Disagree

**There are major aspects of owning a dog I don’t like.**

Strongly agree

Agree

Neither Agree nor Disagree

Disagree

Strongly Disagree

**It bothers me that my dog stops me doing things I enjoyed doing before I owned it**

Strongly agree

Agree

Neither Agree nor Disagree

Disagree

Strongly Disagree

**My dog costs too much money.**

Strongly agree

Agree

Neither Agree nor Disagree

Disagree

Strongly Disagree

**My dog makes too much mess.**

Strongly agree

Agree

Neither Agree nor Disagree

Disagree

Strongly Disagree

**Page 5: Behaviour and Training**

In this section, you will be asked questions about how your dog behaves in different situations as well as how obedient they are in response to training cues.

**Section One: Training and Obedience**

*Some dogs are more obedient and trainable than others. Please indicate how trainable or obedient your dog has been in each of the following situations in the recent past.*

**When off the leash, returns immediately when called.**

Never

Seldom

Sometimes

Usually

Always

Not observed/Not applicable

**Obeys the "sit" command immediately.**

Never

Seldom

Sometimes

Usually

Always

Not observed/Not applicable

**Obeys the "stay" command immediately.**

Never

Seldom

Sometimes

Usually

Always

Not observed/Not applicable

**Seems to attend/listen closely to everything you say or do.**

Never

Seldom

Sometimes

Usually

Always

Not observed/Not applicable

**Slow to respond to correction or punishment; "thick-skinned".**

Never

Seldom

Sometimes

Usually

Always

Not observed/Not applicable

**Slow to learn new tricks or tasks.**

Never

Seldom

Sometimes

Usually

Always

Not observed/Not applicable

**Easily distracted by interesting sights, sounds, or smells.**

Never

Seldom

Sometimes

Usually

Always

Not observed/Not applicable

**Will "fetch" or attempt to fetch sticks, balls, or objects.**

Never

Seldom

Sometimes

Usually

Always

Not observed/Not applicable

**Section Two: Aggression**

*Some dogs display aggressive behaviour from time to time. Typical signs of moderate aggression in dogs include barking, growling and baring teeth. More serious aggression generally includes snapping, lunging, biting, or attempting to bite. Please indicate your own dog's recent tendency to display aggressive behaviour in each of the following contexts:*

**When verbally corrected or punished (scolded, shouted at, etc.) by you or a household member.**

No Aggression - no visible signs of aggression

Moderate Aggression - growling/barking, baring teeth

Serious Aggression - Snaps, bites, or attempts to bite

Not observed/not applicable

**When approached directly by an unfamiliar adult while being walked/exercised on a lead.**

No Aggression - no visible signs of aggression

Moderate Aggression - growling/barking, baring teeth

Serious Aggression - Snaps, bites, or attempts to bite

Not observed/not applicable

**When approached directly by an unfamiliar child while being walked/exercised on a lead.**

No Aggression - no visible signs of aggression

Moderate Aggression - growling/barking, baring teeth

Serious Aggression - Snaps, bites, or attempts to bite

Not observed/not applicable

**Toward unfamiliar persons approaching the dog while s/he is in your car (at the petrol station, for example)**

No Aggression - no visible signs of aggression

Moderate Aggression - growling/barking, baring teeth

Serious Aggression - Snaps, bites, or attempts to bite

Not observed/not applicable

**When toys, bones or other objects are taken away by a household member.**

No Aggression - no visible signs of aggression

Moderate Aggression - growling/barking, baring teeth

Serious Aggression - Snaps, bites, or attempts to bite

Not observed/not applicable

**When bathed or groomed by a household member.**

No Aggression - no visible signs of aggression

Moderate Aggression - growling/barking, baring teeth

Serious Aggression - Snaps, bites, or attempts to bite

Not observed/not applicable

**When an unfamiliar person approaches you or another member of your family at home.**

No Aggression - no visible signs of aggression

Moderate Aggression - growling/barking, baring teeth

Serious Aggression - Snaps, bites, or attempts to bite

Not observed/not applicable

**When unfamiliar persons approach you or another member of your family away from your home.**

No Aggression - no visible signs of aggression

Moderate Aggression - growling/barking, baring teeth

Serious Aggression - Snaps, bites, or attempts to bite

Not observed/not applicable

**When approached directly by a household member while s/he is eating.**

No Aggression - no visible signs of aggression

Moderate Aggression - growling/barking, baring teeth

Serious Aggression - Snaps, bites, or attempts to bite

Not observed/not applicable

**When mailmen or other delivery workers approach your home.**

No Aggression - no visible signs of aggression

Moderate Aggression - growling/barking, baring teeth

Serious Aggression - Snaps, bites, or attempts to bite

Not observed/not applicable

**When his/her food is taken away by a household member.**

No Aggression - no visible signs of aggression

Moderate Aggression - growling/barking, baring teeth

Serious Aggression - Snaps, bites, or attempts to bite

Not observed/not applicable

**When strangers walk past your home while your dog is outside or in the yard.**

No Aggression - no visible signs of aggression

Moderate Aggression - growling/barking, baring teeth

Serious Aggression - Snaps, bites, or attempts to bite

Not observed/not applicable

**When an unfamiliar person tries to touch or pet the dog.**

No Aggression - no visible signs of aggression

Moderate Aggression - growling/barking, baring teeth

Serious Aggression - Snaps, bites, or attempts to bite

Not observed/not applicable

**When joggers, cyclists, rollerbladers or skateboarders pass your home while your dog is outside or in the yard.**

No Aggression - no visible signs of aggression

Moderate Aggression - growling/barking, baring teeth

Serious Aggression - Snaps, bites, or attempts to bite

Not observed/not applicable

**When approached directly by an unfamiliar male dog while being walked/exercised on a lead.**

No Aggression - no visible signs of aggression

Moderate Aggression - growling/barking, baring teeth

Serious Aggression - Snaps, bites, or attempts to bite

Not observed/not applicable

**When approached directly by an unfamiliar female dog while being walked/exercised on a lead.**

No Aggression - no visible signs of aggression

Moderate Aggression - growling/barking, baring teeth

Serious Aggression - Snaps, bites, or attempts to bite

Not observed/not applicable

**When stared at directly by a member of the household.**

No Aggression - no visible signs of aggression

Moderate Aggression - growling/barking, baring teeth

Serious Aggression - Snaps, bites, or attempts to bite

Not observed/not applicable

**Toward unfamiliar dogs visiting your home.**

No Aggression - no visible signs of aggression

Moderate Aggression - growling/barking, baring teeth

Serious Aggression - Snaps, bites, or attempts to bite

Not observed/not applicable

**Toward cats, squirrels or other animals entering your yard.**

No Aggression - no visible signs of aggression

Moderate Aggression - growling/barking, baring teeth

Serious Aggression - Snaps, bites, or attempts to bite

Not observed/not applicable

**Toward unfamiliar persons visiting your home.**

No Aggression - no visible signs of aggression

Moderate Aggression - growling/barking, baring teeth

Serious Aggression - Snaps, bites, or attempts to bite

Not observed/not applicable

**When barked, growled, or lunged at by another (unfamiliar) dog.**

No Aggression - no visible signs of aggression

Moderate Aggression - growling/barking, baring teeth

Serious Aggression - Snaps, bites, or attempts to bite

Not observed/not applicable

**When stepped over by a member of the household.**

No Aggression - no visible signs of aggression

Moderate Aggression - growling/barking, baring teeth

Serious Aggression - Snaps, bites, or attempts to bite

Not observed/not applicable

**When you or a household member retrieves food or objects stolen by the dog.**

No Aggression - no visible signs of aggression

Moderate Aggression - growling/barking, baring teeth

Serious Aggression - Snaps, bites, or attempts to bite

Not observed/not applicable

**Towards another (familiar) dog in your household.**

No Aggression - no visible signs of aggression

Moderate Aggression - growling/barking, baring teeth

Serious Aggression - Snaps, bites, or attempts to bite

Not observed/not applicable

**When approached at a favourite resting/sleeping place by another (familiar) household dog.**

No Aggression - no visible signs of aggression

Moderate Aggression - growling/barking, baring teeth

Serious Aggression - Snaps, bites, or attempts to bite

Not observed/not applicable

**When approached while eating by another (familiar) household dog.**

No Aggression - no visible signs of aggression

Moderate Aggression - growling/barking, baring teeth

Serious Aggression - Snaps, bites, or attempts to bite

Not observed/not applicable

**When approached while playing with/chewing a favourite toy, bone, object, etc., by another (familiar) household dog.**

No Aggression - no visible signs of aggression

Moderate Aggression - growling/barking, baring teeth

Serious Aggression - Snaps, bites, or attempts to bite

Not observed/not applicable

**Section 3: Fear and Anxiety**

*Dogs sometimes show signs of anxiety or fear when exposed to particular sounds, objects, persons or situations. Typical signs of mild to moderate fear include: avoiding eye contact, avoidance of the feared object, crouching or cringing with tail lowered or tucked between the legs, whimpering and whining, freezing, and shaking and trembling. Extreme fear is characterized by exaggerated cowering, and/or vigorous attempts to escape, retreat or hide from the feared object, person or situation. Please indicate your own dog's recent tendency to display fearful behaviour in each of the following contexts:*

**When approached directly by an unfamiliar adult while away from your home.**

No fear or Anxiety - no visible signs of fear

Mild-Moderate fear/anxiety

Extreme Fear - cowers, retreats, hides, etc

Not observed/not applicable

**When approached directly by an unfamiliar child while away from your home.**

No fear or Anxiety - no visible signs of fear

Mild-Moderate fear/anxiety

Extreme Fear - cowers, retreats, hides, etc

Not observed/not applicable

**In response to sudden or loud noises (e.g. vacuum cleaner, car backfire, road drills, objects being dropped, etc.).**

No fear or Anxiety - no visible signs of fear

Mild-Moderate fear/anxiety

Extreme Fear - cowers, retreats, hides, etc

Not observed/not applicable

**When unfamiliar persons visit your home.**

No fear or Anxiety - no visible signs of fear

Mild-Moderate fear/anxiety

Extreme Fear - cowers, retreats, hides, etc

Not observed/not applicable

**When an unfamiliar person tries to touch or pet the dog.**

No fear or Anxiety - no visible signs of fear

Mild-Moderate fear/anxiety

Extreme Fear - cowers, retreats, hides, etc

Not observed/not applicable

**In heavy traffic.**

No fear or Anxiety - no visible signs of fear

Mild-Moderate fear/anxiety

Extreme Fear - cowers, retreats, hides, etc

Not observed/not applicable

**In response to strange or unfamiliar objects on or near the path (e.g. plastic rubbish bags, leaves, litter, flags flapping, etc.).**

No fear or Anxiety - no visible signs of fear

Mild-Moderate fear/anxiety

Extreme Fear - cowers, retreats, hides, etc

Not observed/not applicable

**When examined/treated by a veterinarian.**

No fear or Anxiety - no visible signs of fear

Mild-Moderate fear/anxiety

Extreme Fear - cowers, retreats, hides, etc

Not observed/not applicable

**During thunderstorms, firework displays, or similar events.**

No fear or Anxiety - no visible signs of fear

Mild-Moderate fear/anxiety

Extreme Fear - cowers, retreats, hides, etc

Not observed/not applicable

**When approached directly by an unfamiliar dog of the same or larger size.**

No fear or Anxiety - no visible signs of fear

Mild-Moderate fear/anxiety

Extreme Fear - cowers, retreats, hides, etc

Not observed/not applicable

**When approached directly by an unfamiliar dog of smaller size.**

No fear or Anxiety - no visible signs of fear

Mild-Moderate fear/anxiety

Extreme Fear - cowers, retreats, hides, etc

Not observed/not applicable

**When first exposed to unfamiliar situations (e.g. first car trip, first time in elevator, first visit to veterinarian, etc.)**

No fear or Anxiety - no visible signs of fear

Mild-Moderate fear/anxiety

Extreme Fear - cowers, retreats, hides, etc

Not observed/not applicable

**In response to wind or wind-blown objects.**

No fear or Anxiety - no visible signs of fear

Mild-Moderate fear/anxiety

Extreme Fear - cowers, retreats, hides, etc

Not observed/not applicable

**When having nails clipped by a household member.**

No fear or Anxiety - no visible signs of fear

Mild-Moderate fear/anxiety

Extreme Fear - cowers, retreats, hides, etc

Not observed/not applicable

**When groomed or bathed by a household member.**

No fear or Anxiety - no visible signs of fear

Mild-Moderate fear/anxiety

Extreme Fear - cowers, retreats, hides, etc

Not observed/not applicable

**When having his/her feet towelled by a member of the household.**

No fear or Anxiety - no visible signs of fear

Mild-Moderate fear/anxiety

Extreme Fear - cowers, retreats, hides, etc

Not observed/not applicable

**When unfamiliar dogs visit your home.**

No fear or Anxiety - no visible signs of fear

Mild-Moderate fear/anxiety

Extreme Fear - cowers, retreats, hides, etc

Not observed/not applicable

**When barked, growled, or lunged at by an unfamiliar dog.**

No fear or Anxiety - no visible signs of fear

Mild-Moderate fear/anxiety

Extreme Fear - cowers, retreats, hides, etc

Not observed/not applicable

**Section 4: Separation-related behaviour**

*Some dogs show signs of anxiety or abnormal behaviour when left alone, even for relatively short periods of time. Thinking back over the recent past, how often has your dog shown each of the following signs of separation-related behaviour when left, or about to be left, on its own:*

**Shaking, shivering, or trembling.**

Never

Seldom

Sometimes

Usually

Always

Not applicable

**Excessive salivation.**

Never

Seldom

Sometimes

Usually

Always

Not applicable

**Restlessness, agitation, or pacing.**

Never

Seldom

Sometimes

Usually

Always

Not applicable

**Whining**.

Never

Seldom

Sometimes

Usually

Always

Not applicable

**Barking**.

Never

Seldom

Sometimes

Usually

Always

Not applicable

**Howling.**

Never

Seldom

Sometimes

Usually

Always

Not applicable

**Chewing or scratching at doors, floor, windows, curtains, etc.**

Never

Seldom

Sometimes

Usually

Always

Not applicable

**Loss of appetite.**

Never

Seldom

Sometimes

Usually

Always

Not applicable

**Section 5: Excitability**

*Some dogs show relatively little reaction to sudden or potentially exciting events and disturbances in their environment, while others become highly excited at the slightest novelty. Signs of mild to moderate excitability include increased alertness, movement toward the source of novelty, and brief episodes of barking. Extreme excitability is characterized by a general tendency to over-react. The excitable dog barks or yelps hysterically at the slightest disturbance, rushes toward and around any source of excitement, and is difficult to calm down. Please indicate your own dog's recent tendency to become excitable in each of the following contexts:*

**When you or other members of the household come home after a brief absence.**

Calm - little or no special reaction

Mild - moderate Excitability

Extremely Excitable - over-reacts, hard to calm down

Not/observed/not applicable

**When playing with you or other members of your household.**

Calm - little or no special reaction

Mild - moderate Excitability

Extremely Excitable - over-reacts, hard to calm down

Not/observed/not applicable

**When doorbell rings.**

Calm - little or no special reaction

Mild - moderate Excitability

Extremely Excitable - over-reacts, hard to calm down

Not/observed/not applicable

**Just before being taken for a walk.**

Calm - little or no special reaction

Mild - moderate Excitability

Extremely Excitable - over-reacts, hard to calm down

Not/observed/not applicable

**Just before being taken on a car trip.**

Calm - little or no special reaction

Mild - moderate Excitability

Extremely Excitable - over-reacts, hard to calm down

Not/observed/not applicable

**When visitors arrive at your home.**

Calm - little or no special reaction

Mild - moderate Excitability

Extremely Excitable - over-reacts, hard to calm down

Not/observed/not applicable

**Section 6: Attachment and attention-seeking**

*Most dogs are strongly attached to their people, and some demand a great deal of attention and affection from them. Thinking back over the recent past, how often has your dog shown each of the following signs of attachment or attention-seeking:*

**Displays a strong attachment for one particular member of the household.**

Never

Seldom

Sometimes

Usually

Always

Not observed/not applicable

**Tends to follow you (or other members of the household) about the house, from room to room.**

Never

Seldom

Sometimes

Usually

Always

Not observed/not applicable

**Tends to sit close to, or in contact with, you (or others) when you are sitting down.**

Never

Seldom

Sometimes

Usually

Always

Not observed/not applicable

**Tends to nudge, nuzzle or paw you (or others) for attention when you are sitting down.**

Never

Seldom

Sometimes

Usually

Always

Not observed/not applicable

**Becomes agitated (whines, jumps up, tries to intervene) when you (or others) show affection for another person.**

Never

Seldom

Sometimes

Usually

Always

Not observed/not applicable

**Becomes agitated (whines, jumps up, tries to intervene) when you (or others) show affection for another dog or animal.**

Never

Seldom

Sometimes

Usually

Always

Not observed/not applicable

**Section 7**

*Dogs display a wide range of miscellaneous behaviour problems in addition to those already covered by this questionnaire. Thinking back over the recent past, please indicate how often your dog has shown any of the following behaviours:*

**Chases or would chase cats given the opportunity.**

Never

Seldom

Sometimes

Usually

Always

Not observed/not applicable

**Chases or would chase birds given the opportunity.**

Never

Seldom

Sometimes

Usually

Always

Not observed/not applicable

**Chases or would chase squirrels, rabbits and other small animals given the opportunity.**

Never

Seldom

Sometimes

Usually

Always

Not observed/not applicable

**Escapes or would escape from home or yard given the chance.**

Never

Seldom

Sometimes

Usually

Always

Not observed/not applicable

**Rolls in animal droppings or other "smelly" substances.**

Never

Seldom

Sometimes

Usually

Always

Not observed/not applicable

**Eats own or other animals' droppings or faeces.**

Never

Seldom

Sometimes

Usually

Always

Not observed/not applicable

**Chews inappropriate objects.**

Never

Seldom

Sometimes

Usually

Always

Not observed/not applicable

**"Mounts" objects, furniture, or people.**

Never

Seldom

Sometimes

Usually

Always

Not observed/not applicable

**Begs persistently for food when people are eating.**

Never

Seldom

Sometimes

Usually

Always

Not observed/not applicable

**Steals food.**

Never

Seldom

Sometimes

Usually

Always

Not observed/not applicable

**Nervous or frightened on stairs.**

Never

Seldom

Sometimes

Usually

Always

Not observed/not applicable

**Pulls excessively hard when on the leash.**

Never

Seldom

Sometimes

Usually

Always

Not observed/not applicable

**Urinates against objects/furnishings in your home.**

Never

Seldom

Sometimes

Usually

Always

Not observed/not applicable

**Urinates when approached, petted, handled or picked up.**

Never

Seldom

Sometimes

Usually

Always

Not observed/not applicable

**Urinates when left alone at night, or during the daytime.**

Never

Seldom

Sometimes

Usually

Always

Not observed/not applicable

**Defecates when left alone at night, or during the daytime.**

Never

Seldom

Sometimes

Usually

Always

Not observed/not applicable

**Hyperactive, restless, has trouble settling down.**

Never

Seldom

Sometimes

Usually

Always

Not observed/not applicable

**Playful, puppyish, boisterous.**

Never

Seldom

Sometimes

Usually

Always

Not observed/not applicable

**Active, energetic, always on the go.**

Never

Seldom

Sometimes

Usually

Always

Not observed/not applicable

**Becomes highly excited/distracted when encountering other (unfamiliar) dogs:**

Never

Seldom

Sometimes

Usually

Always

Not observed/not applicable

**Becomes highly excited/distracted when encountering other (unfamiliar) people:**

Never

Seldom

Sometimes

Usually

Always

Not observed/not applicable

**When out and about, is easily distracted or preoccupied by smells (i.e., engages in persistent sniffing of ground or objects):**

Never

Seldom

Sometimes

Usually

Always

Not observed/not applicable

**Has difficulty shifting attention away from interesting or distracting stimuli (e.g., other dogs, smells, people, small animals, etc):**

Never

Seldom

Sometimes

Usually

Always

Not observed/not applicable

**Is slow to recover after being startled or frightened (appears anxious/fearful long after the event):**

Never

Seldom

Sometimes

Usually

Always

Not observed/not applicable

**Stares intently at nothing visible.**

Never

Seldom

Sometimes

Usually

Always

Not observed/not applicable

**Snaps at (invisible) flies.**

Never

Seldom

Sometimes

Usually

Always

Not observed/not applicable

**Chases own tail/hind end.**

Never

Seldom

Sometimes

Usually

Always

Not observed/not applicable

**Chases/follows shadows, light spots, etc.**

Never

Seldom

Sometimes

Usually

Always

Not observed/not applicable

**Barks persistently when alarmed or excited.**

Never

Seldom

Sometimes

Usually

Always

Not observed/not applicable

**Licks him/herself excessively.**

Never

Seldom

Sometimes

Usually

Always

Not observed/not applicable

**Licks people or objects excessively.**

Never

Seldom

Sometimes

Usually

Always

Not observed/not applicable

**Displays other bizarre, strange, or repetitive behaviour(s)**

Never

Seldom

Sometimes

Usually

Always

Not observed/not applicable

**Compared to my expectations when I first acquired my dog, training and ongoing maintenance of their basic obedience has been...**

*N.B. If you would like to explain your answer further in your own words, please feel free to do so below.*

1. Harder than I expected
2. As I expected
3. Easier than I expected
4. I’m not sure/I can’t remember

Free text:

**Compared to my expectations when I first acquired my dog, their behaviour in the last three months has been...**

N.B. If you would like to explain your answer further in your own words, please feel free to do so below.

1. Worse than I expected
2. As I expected
3. Better than I expected
4. I’m not sure/I can’t remember

Free text:

**Have you or anyone in your household ever used any of the following aids or methods on/with your dog, to try and change any aspect of their behaviour? Please select all options that apply.**

**[Columns]:**

Yes

1. No

**[Rows]:**

1. Allowing sniffing time/interaction with the environment
2. Anti-bark ‘Husher’ muzzle
3. Bark-activated citronella/vibration/ultrasonic collar
4. Choke/check chain
5. Clicker training
6. Electric collar
7. Electronic boundary fence
8. Food/treats
9. Harness
10. Head collar
11. Lead corrections (e.g., quickly yanking back on/jerking the lead if your dog pulls)
12. Pet Corrector™
13. Physical correction (e.g., smacking, tapping their nose, hitting, pinching)
14. Physical touch, e.g., stroking or patting
15. Physically moving your dog (e.g., pushing on your dog’s hindquarters to get them into a sit, pushing them off furniture or if they jump up at you)
16. Playing with other dogs
17. Playing with you/another household member
18. Prong collar
19. Rattle bottle/cans/discs as a distractor
20. Rubbing their nose in faeces/urine if they toilet in an inappropriate location
21. Shouting at them or telling them off
22. “Time out”: shutting your dog away within the home, away from people (e.g., in another room, in their crate)
23. “Time out”: shutting your dog outdoors (e.g., in the garden/yard or an outside kennel)
24. Slip lead
25. Toys
26. Verbal praise
27. Water pistol/spray bottle

**Have you ever bought any of the following as a way to control your dog’s location either indoors and outdoors? (please select all that apply)**

Playpen

Crate

Dog gates and/or Stair-gates

Tethering/tie-out equipment

Long line for indoors

GPS or trackers

Electric boundary fence?

Dog door

Outdoor kennels

Indoor kennels

**Have you ever used any of the following resources for advice regarding your dog’s behaviour and training?** Please select **all options** that apply

None of these options

The Kennel Club website

An animal charity website, e.g. Dogs Trust, RSPCA, PDSA, etc.

A breed/crossbreed-specific online resource (e.g. website/forum)

Social media sites, e.g. Facebook, Instagram

Book(s)

Dog-specific magazine(s)

My veterinary professional (e.g. veterinary surgeon, veterinary nurse)

Dog trainer (not a behaviourist)

Talking to friends or family who own or had owned a dog

Talking to a dog breeder

I can’t remember

Other (please specify) **[free text]**

**Have you ever *sought* advice from a behaviour professional regarding your dog’s behaviour?**

Yes [logic - goes to a)

No [logic - goes to Part 5]

No but I am thinking about it

**a)Please select who you sought advice from?**

Veterinary Surgeon

Veterinary Nurse

Dog behaviourist

Dog trainer

Dog breeder

All of the above

Other source

I can’t remember

**b) How old was your dog when you first sought advice?**

Less than 3 months old

3 - 5 months old

6 - 12 months old

13 - 24 months old

Over 24 months old

I’m not sure/can’t remember

**b) How much have you spent to date on professional help for your dog’s behaviour?**

< £100

£100 - £200

£201 - £400

£401 - £600

>£600

I’m not sure

***To complete if you have extra time***

**Please read through the following sentences regarding your satisfaction with the purchase of your dog breed and answer using the 5-point scale:**

***I am satisfied with my decision***

Completely disagree

disagree

Neither agree nor disagree

agree

Completely agree

***I am confident I made a deliberate decision***

Completely disagree

disagree

Neither agree nor disagree

agree

Completely agree

***It would have been useful if I had asked for more advice before acquiring my dog***

Completely disagree

disagree

Neither agree nor disagree

agree

Completely agree

***If I were to acquire another dog in the future, I would prepare myself differently***

Completely disagree

disagree

Neither agree nor disagree

agree

Completely agree

***The decision to acquire my dog was the right decision for my current situation***

Completely disagree

disagree

Neither agree nor disagree

agree

Completely agree

***The decision to acquire my dog did not play out as expected***

Completely disagree

disagree

Neither agree nor disagree

agree

Completely agree

***I sometimes have mixed feelings about the decision to acquire my dog***

Completely disagree

disagree

Neither agree nor disagree

agree

Completely agree

**Q. We may wish to get in contact with you in the future for limited reasons outlined below.**

**Please let us know which (if any) you are happy to be contacted about, and provide us with your preferred email address:**

To be asked to clarify my responses to this study

To be sent the results of this study

To be invited to take part in further research about my puppy/dog

None of the above

Comment box: **[email address]**

**Debrief Section**

Thank you very much for taking part in our study, your time and effort in completing these surveys is very much appreciated.

We understand that this survey may have worried some owners who have faced challenges with dog ownership. We have therefore collated some information from some reputable sources below.

**Worried about your dog’s health?**

If you have any concerns about your dog’s health you should contact your vet in the first instance who can examine your dog, take a thorough history and suggest further investigations or treatments that may be necessary. Register with a local vet if you have not yet done so – to find a local veterinary surgeon, please visit the following website:

<https://findavet.rcvs.org.uk/home/>

If you are struggling with the rising cost-of-living, you may meet the eligibility criteria for subsidised/free veterinary services available from charities including the PDSA, RSPCA and Blue Cross:

<https://www.pdsa.org.uk/what-we-do/treat>

<https://www.rspca.org.uk/whatwedo/care/vetcare>

<https://www.bluecross.org.uk/veterinary>

**Worried about your dog’s behaviour?**

If you are worried about a behavioural problem your dog has developed, talk to your vet about being referred to an accredited animal or veterinary behaviourist via the Animal Behaviour and Training Council (ABTC):

<https://abtc.org.uk/practitioners/>

The ABTC also have links to accredited dog trainers across the UK and dog training resources on their website:

<https://abtc.org.uk/owners/>

The COVID-19 pandemic and rising cost-of-living has created challenges for mental health across the UK. If you would like support or advice regarding your own mental health, the following websites may be useful:

<https://www.nhs.uk/oneyou/every-mind-matters/>

<https://www.samaritans.org>
