# Supplementary figures and images for: The doodle dilemma: How the physical health of ‘Designer-crossbreed’ Cockapoo, Labradoodle and Cavapoo dogs’ compares to their purebred progenitor breeds
Source: PLoS One. 2024 Aug 28;19(8):e0306350. doi: 10.1371/journal.pone.0306350 (PMC11355567; doi:10.1371/journal.pone.0306350)

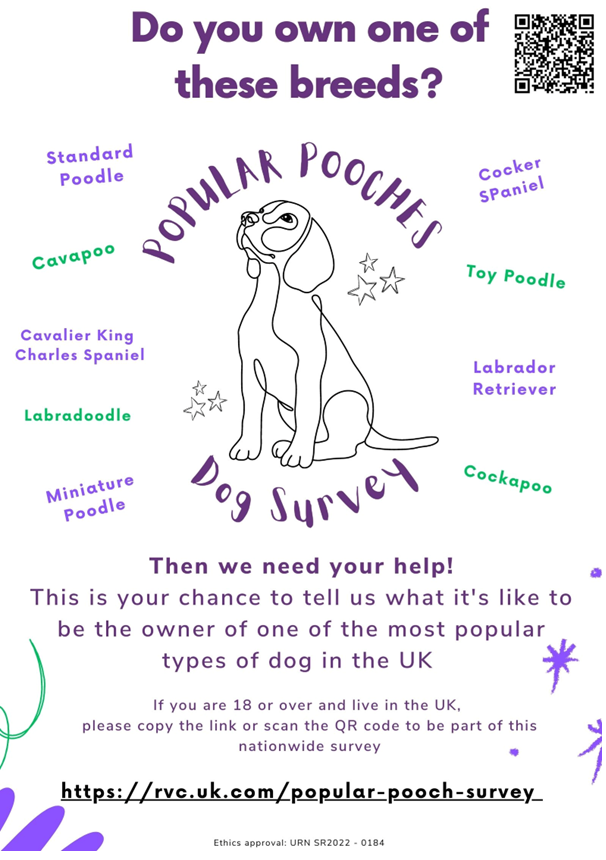


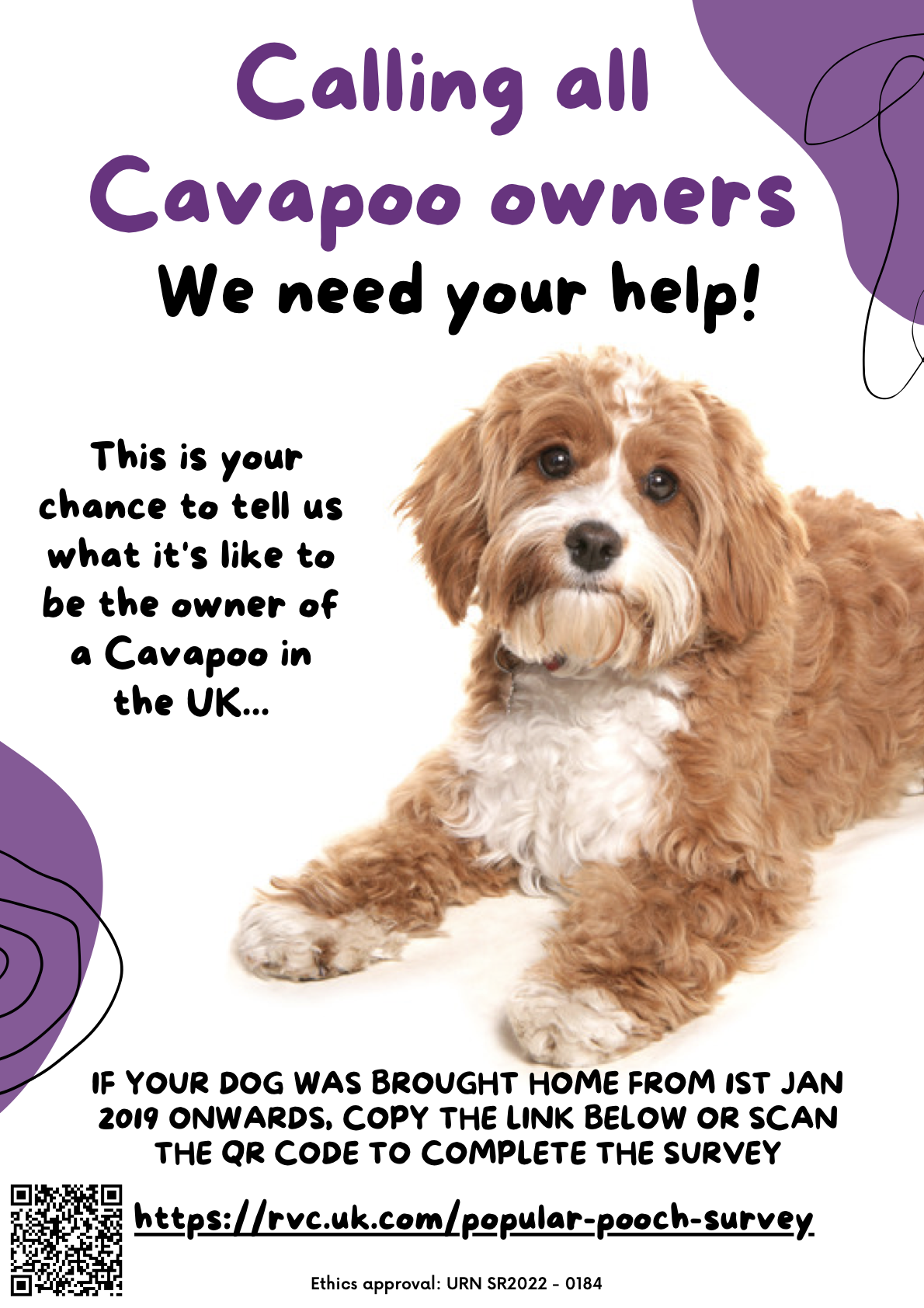


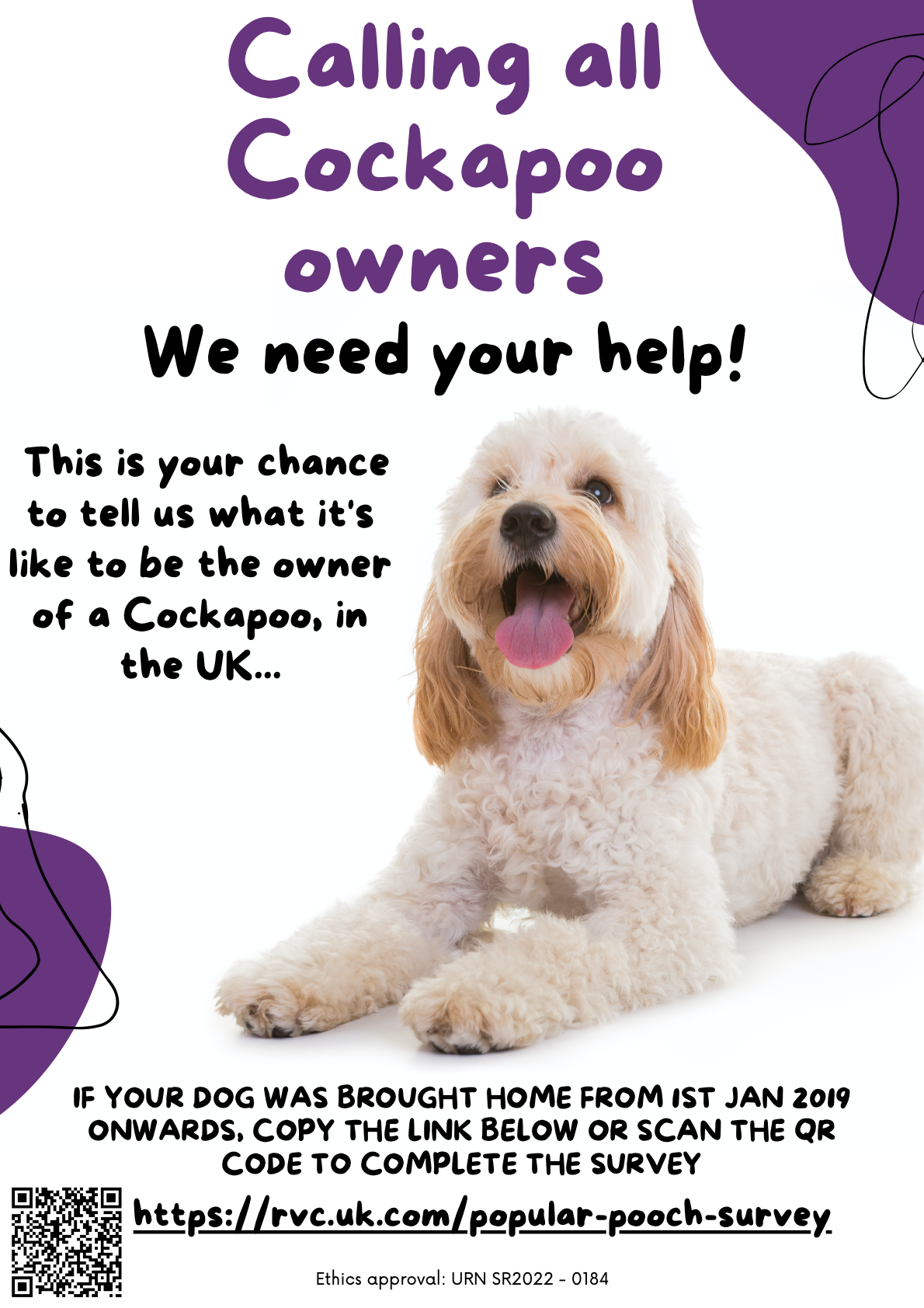


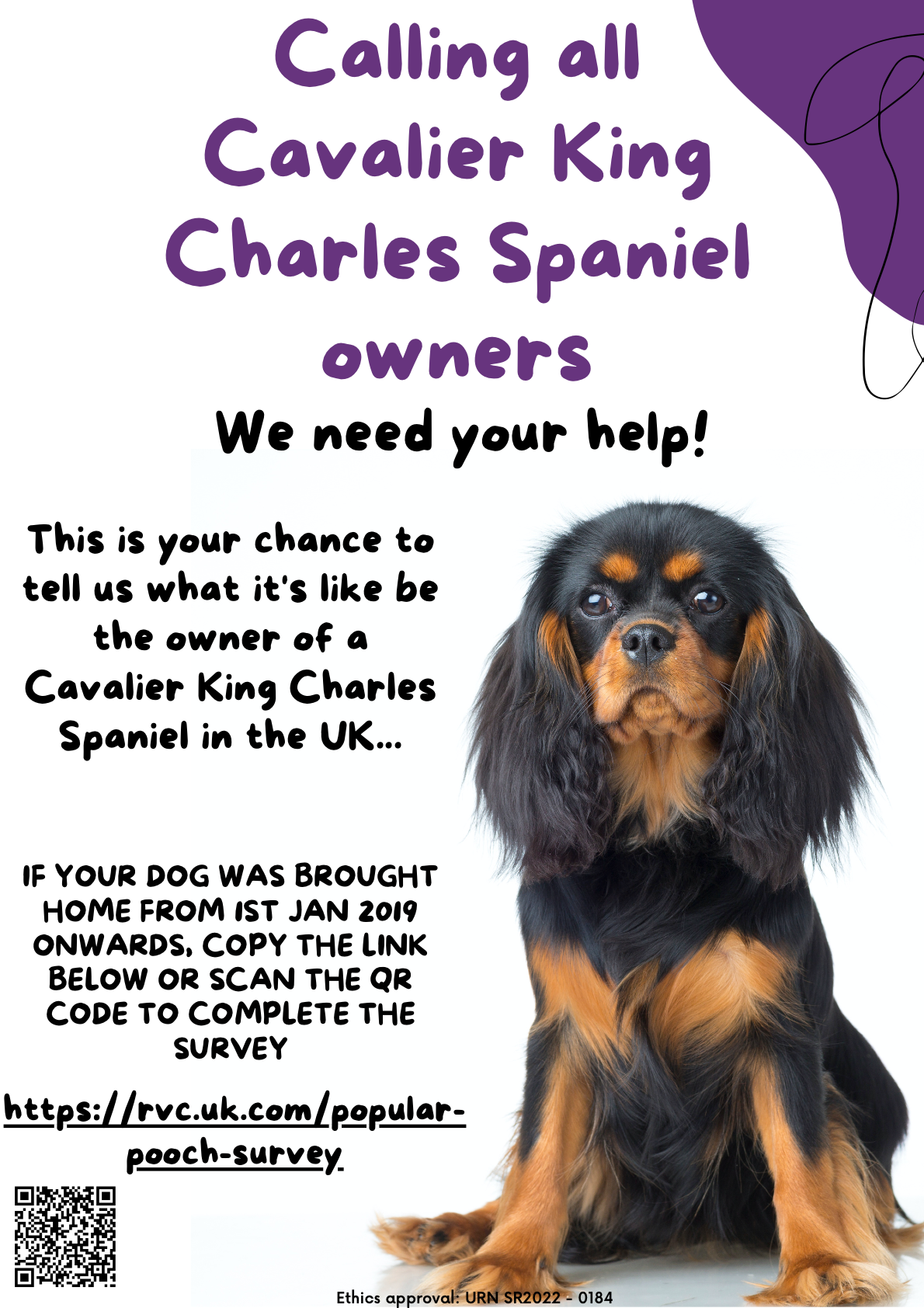


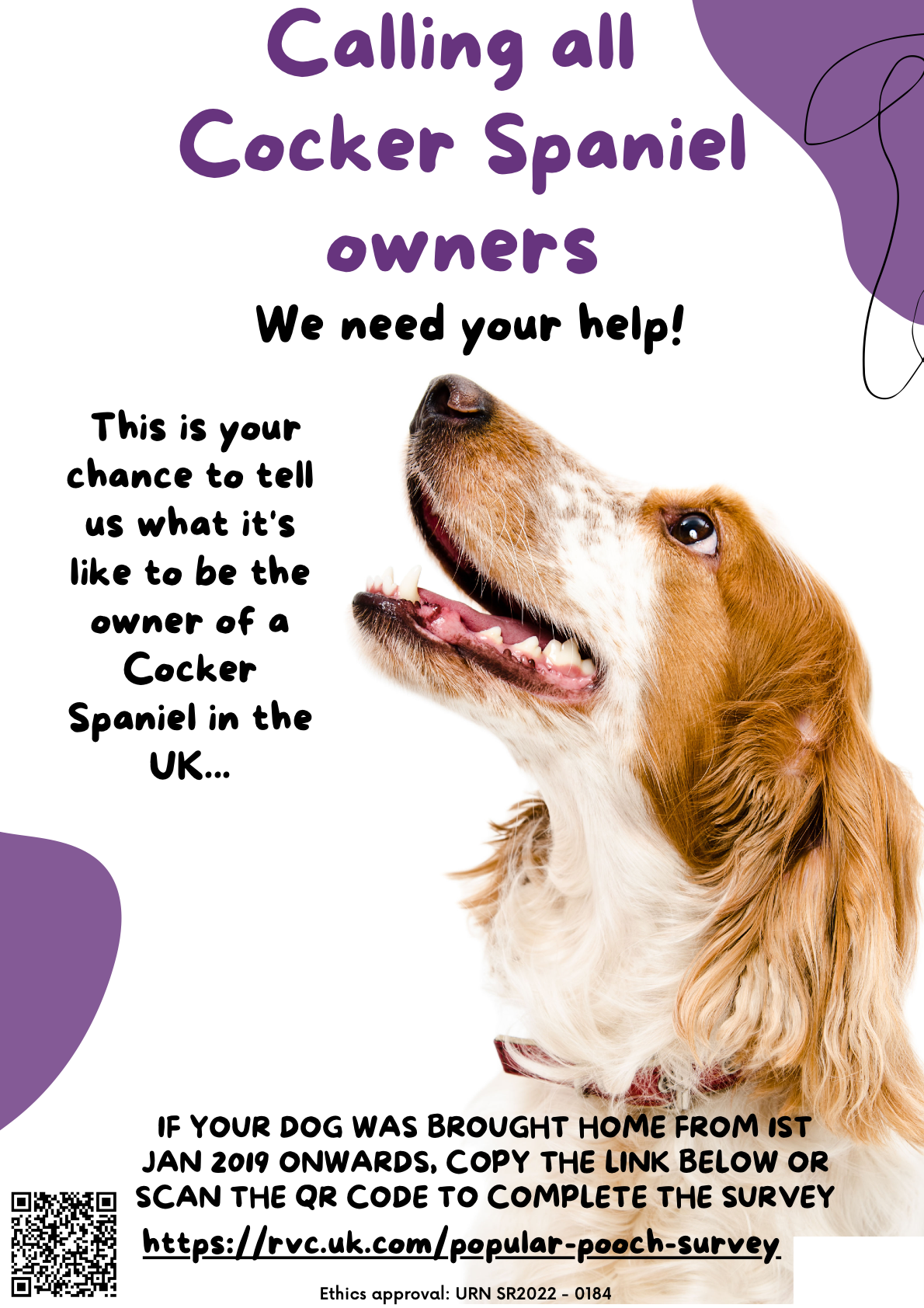


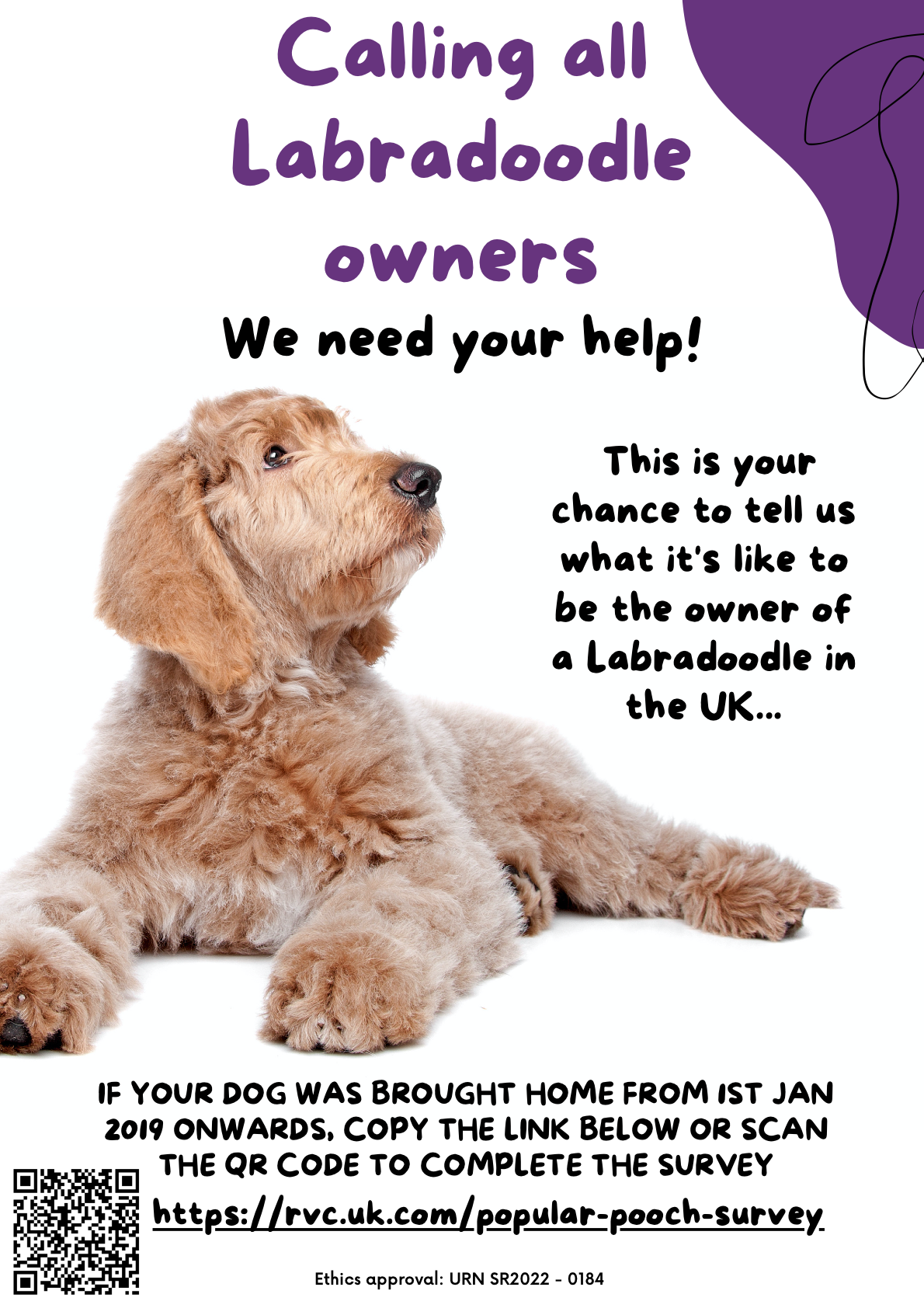


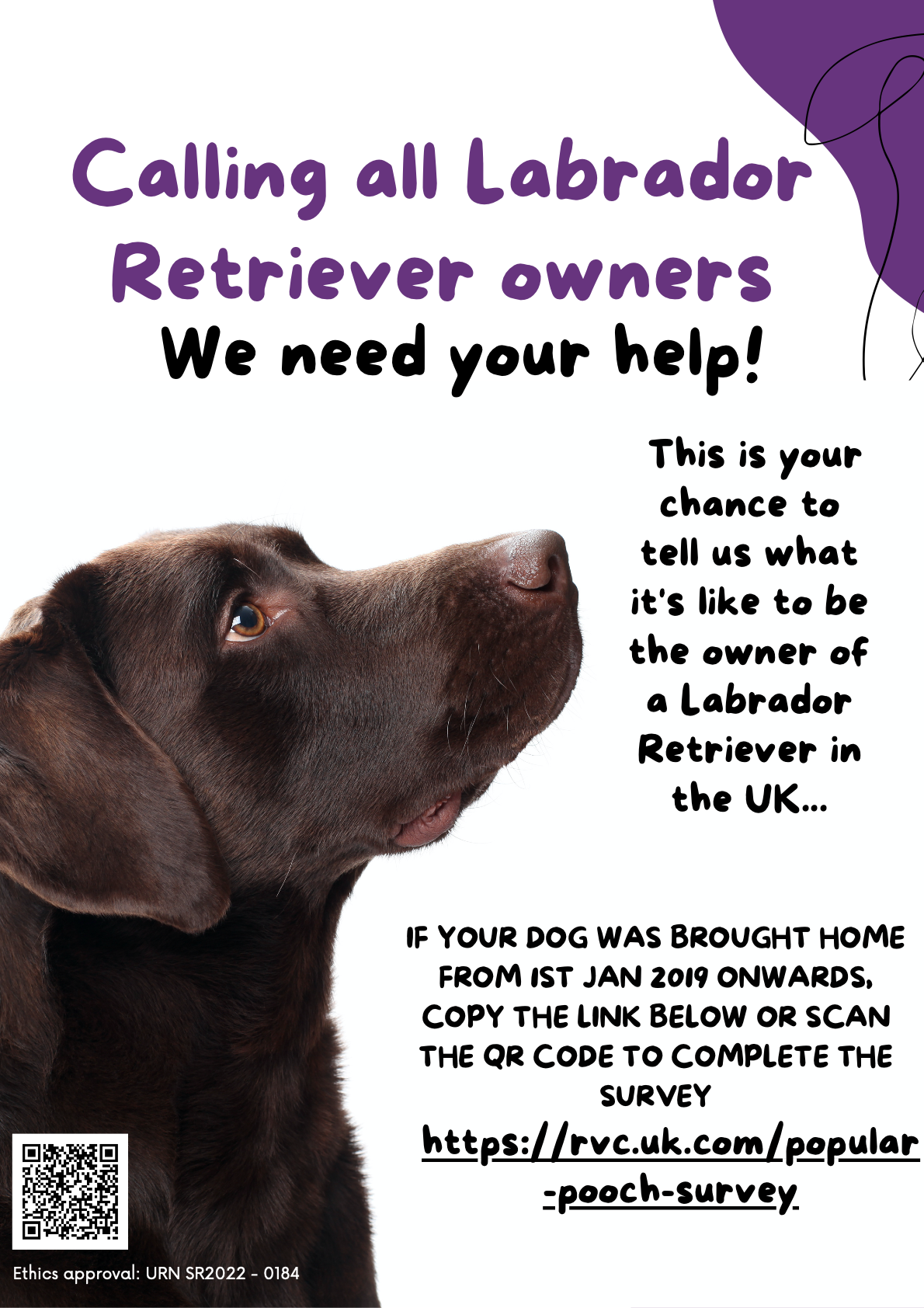


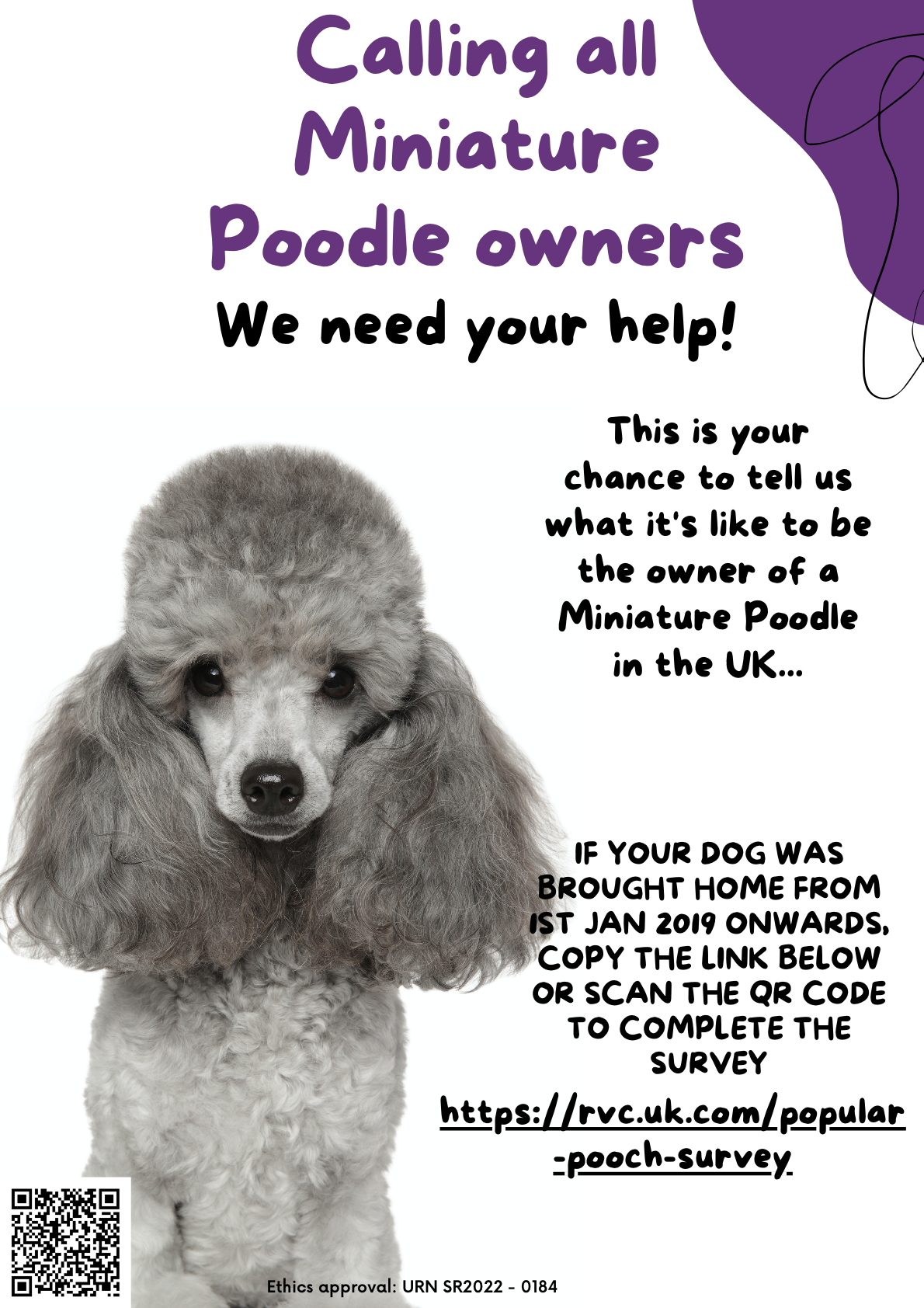


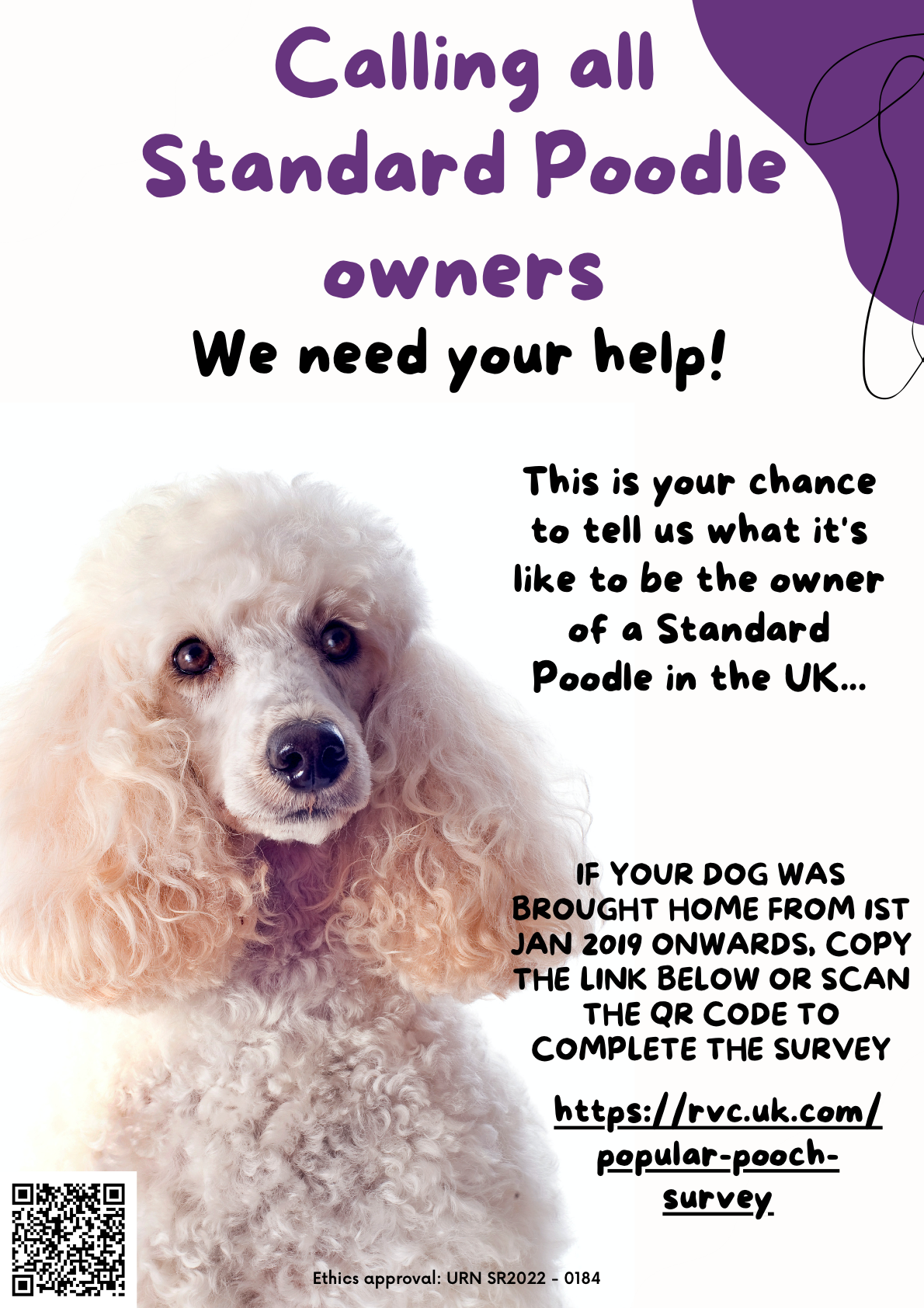


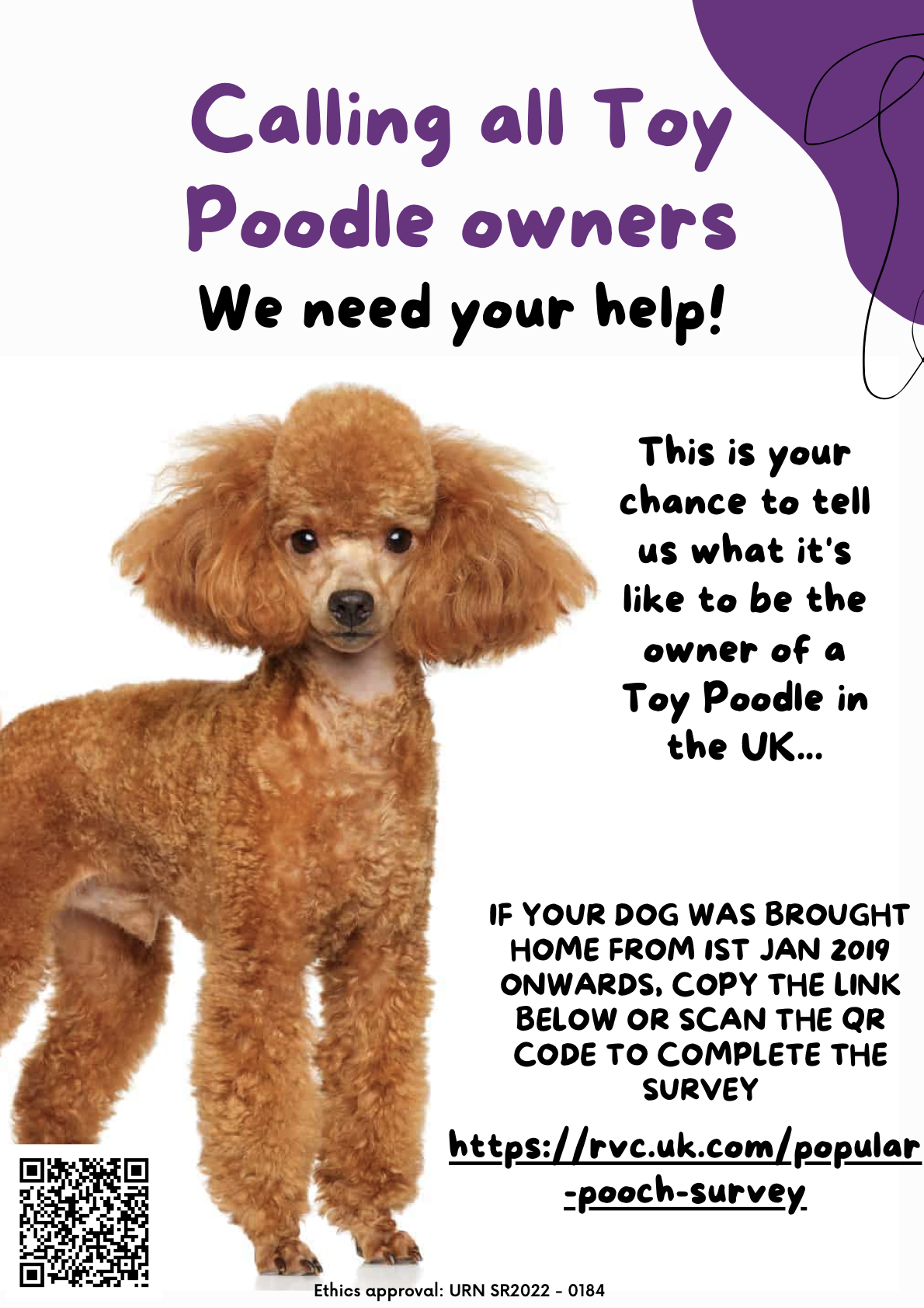

Supplement: S1 Fig — (DOCX) [file pone.0306350.s002.docx]
